# Empowering Adolescents for Sustainable and Inclusive Careers: A Quasi‐Experimental Evaluation of a Life Design‐Based Intervention

**DOI:** 10.1002/jad.70113

**Published:** 2026-01-28

**Authors:** Sara Santilli, Denise Zucchini, Isabella Valbusa, Maria Cristina Ginevra, Terence Tracey, Laura Nota

**Affiliations:** ^1^ Department of Philosophy, Sociology, Education and Applied Psychology University of Padova Padua Italy; ^2^ Society and Multi‐level Governance of Human Right Canter University of Padova Padua Italy; ^3^ Arizona State University's College of Integrative Sciences and Arts Tempe Arizona USA

**Keywords:** career intervention, critical consciousness, decent work, life design, sustainable development

## Abstract

**Background:**

Adolescents must plan their educational and occupational futures in a context shaped by globalization, social inequalities, and environmental crises. Although career guidance increasingly emphasizes sustainable and inclusive development, empirical interventions integrating social justice, sustainability, and critical consciousness within established career frameworks remain limited. Drawing on the life design paradigm and the psychology of working theory, this study evaluates a school‐based career intervention aimed at enhancing career adaptability, perceptions of decent work, and critical consciousness among adolescents.

**Methods:**

Using a quasi‐experimental design, participants were 266 Italian 12th‐grade students (Mage = 17.20 years, SD = 0.50; 77 males, 189 females) attending public high schools in northern Italy. Classes were assigned to an intervention group (*n* = 145) or a no‐treatment control group (*n* = 121). The intervention consisted of 5 weekly 2‐h didactic units delivered during school hours and focused on globalization, technology, inclusion, sustainability, and future career planning. Measures were collected at pre‐test and post‐test for both groups, with a 6‐month follow‐up for the intervention group. Data were analyzed using linear mixed models.

**Results:**

Compared with controls, students in the intervention group showed greater improvements in career adaptability (concern, curiosity, and collaboration), more future‐oriented perceptions of decent work, higher critical consciousness, and increased career decisiveness and internality. Improvements were sustained at 6‐month follow‐up.

**Conclusions:**

Findings support the efficacy of a life design–based career intervention integrating sustainability and social justice, highlighting the potential of career education to foster agency and critical awareness for inclusive and sustainable career development.

Young people reflect on their professional future in a context shaped by interconnected global challenges. These include evolving employment patterns due to globalization and technological advancements, increasing social inequalities, and ecological crises such as climate change and resource depletion (Cohen‐Scali et al. 2018; Makridakis 2017; Guichard 2018; Parola et al. 2022). How can educational interventions equip young people to address these challenges through sustainable and inclusive career planning?

Recent meta‐analyses (Brown et al. 2003) have emphasized the “critical ingredients” necessary for effective career interventions, such as individualized feedback, modeling, and action planning. Our program includes many of these features, such as structured group discussions and personalized reflection. Furthermore, this study draws upon the Psychology of Working Theory (PWT; Blustein 2006; Duffy et al. 2016), which highlights the roles of marginalization, access to decent work, and critical agency in vocational development. The integration of PWT and Life Design (Savickas et al. 2009) offers a novel framework for empowering youth in facing systemic challenges (Guichard 2022).

Considering these global challenges, the field of career guidance has shown increasing but still limited attention to critical consciousness, decent work, and environmental and social sustainability (Rochat and Rossier 2023; Carosin et al. 2022; Cadenas and McWhirter 2022a). Career guidance can play a fundamental role in promoting critical consciousness in young people by addressing contextual challenges that impact people and the environment, fostering ideas about decent work, and encouraging professional choices that contribute to environmental protection and societal well‐being (Cohen‐Scali et al. 2018; Guichard 2022; Cadenas and McWhirter 2022a).

Within the Life Design paradigm, which is based on the epistemology of social constructionism, particular attention is given to preparing youth for their professional futures. This involves encouraging reflection on their lives and the broader global challenges, as well as emphasizing professional choices that promote equitable and sustainable development (Guichard 2022; Pouyaud and Guichard 2017).

However, as highlighted by Guichard (2022), there is a lack of practical and research contributions in this area. To address this gap, the present study develops and evaluates a career education program aimed at promoting a nuanced understanding of decent work (i.e., work that protects rights, inclusion, and sustainability) and strengthening critical consciousness (i.e., awareness of structural social and environmental inequalities and agency to respond to them).

This program seeks to address contextual challenges that may serve as barriers to professional and personal growth, while encouraging career choices that consider global, ecological, and human needs.

## 1 Life Design for an Inclusive and Sustainable Future

Based on the self‐construction perspective (Guichard 2005) and social constructionism (Savickas 2011), the Life Design paradigm was developed during the period of the economic crisis at the end of the first decade of the 2000s to detect solutions to the problems generated by the global changes occurring, underlining the increasing importance of promoting access to a dignified life and job for everyone (Chhabra et al. 2022). This shift was driven by the recognition that traditional career trajectories, which were once linear and predictable, had become fragmented and uncertain. As a result, the previous approach to career development was no longer applicable, making a paradigm shift essential to better address the complex and nonlinear nature of modern work life.

As a new career counseling paradigm, Life Design goes beyond merely providing professional information, matching individuals with employer expectations, and training them in job search activities. Instead, it emphasizes supporting individuals in a broader life design process, taking into account not only their professional roles but also other significant roles and themes in their lives. By fostering reflection and developing a new critical consciousness of the self and the world, life design encourages individuals to explore their identities, values, and aspirations holistically, leading to a more integrated and fulfilling approach to both career and personal life (Drabik‐Podgórna and Makowska 2022).

As a consequence, interventions based on Life Design are not focused on the definition of the best ways to choose a job, find it, or adapt to the demands of the labor market. These interventions aim at preparing young people for their future careers, making them adapt to career transitions and challenges, and the complexity of the labor market (Nota and Rossier 2015). This means educating young people about the processes that shape the labor market and social context, while providing them with the skills and resources needed to successfully navigate the changes in the world of work (Nota and Rossier 2015).

Life design paradigm is therefore particularly focused on several constructs such as career adaptability, a positive view of the future, and career decidedness, useful for adolescents in positively projecting themselves toward their own future and career planning (Savickas et al. 2009; Ginevra et al. 2017). Among these, career adaptability is considered the fundamental construct of the life design paradigm (Savickas et al. 2009). In addition, identity construction represents a central process within the life design paradigm, particularly during adolescence. Life Design interventions explicitly support individuals in developing coherent vocational identities that foster a sense of self‐continuity, meaning, and agency across educational and occupational transitions, especially in contexts characterized by instability and uncertainty (Savickas et al. 2009).

Career adaptability is a psychosocial construct that refers to the resources a person has to cope with assignments, transitions, and traumas (difficulties?) in their career (Savickas 2011). The dimensions of career adaptability are known as the 4Cs: (1) concern is the propensity to worry positively about the future; (2) control is the conviction that the future is at least partially manageable; (3) curiosity is the openness toward the professional world; (4) confidence is referred to the beliefs of the self to achieve one's career goals and solve problems (Savickas 1997). An important fifth component is collaboration (Nota et al. 2012), that emphasizes the importance of working effectively with others, building networks, and engaging in teamwork. In this dimension is recognized that career success depends not only on individual capabilities but also on the ability to cooperate with others, share knowledge, and contribute to collective goals. This collaborative mindset is essential for navigating the complexities of modern work environments and fostering professional growth.

Previous literature has analyzed the consequences of being career adapt‐able in young people, proving that increased professional adaptability is connected to a higher ability to overcome obstacles, the tendency to take into consideration systemic challenges to reach sustainable development, relying on more personal resources, as optimism, hope, persistence, the ability to look for and handle information, and the ability to picture different future circumstances (Creed et al. 2009; Hirschi 2009; Maggiori et al. 2013; Di Maggio et al. 2020). Career adaptability is also related to a broad range of positive career outcomes, such as career decision‐making, career satisfaction, etc., emphasizing its relevance to coping with the various transitions that people deal with through their professional lives (Rudolph et al. 2017).

In recent years, the life design paradigm has focused on the importance of futures based on inclusion, sustainability, and social justice in career and life interventions. Related to this, particular attention is focused on new dimensions in career guidance, such as decent work, critical consciousness, personal and social reflexivity, the tendency to consider systemic challenges to achieve sustainable development, cosmopolitanism, etc. (Guichard 2022; Nota et al. 2020). In this paper, we focus on decent work and critical consciousness.

## 2 Decent Work for an Inclusive and Sustainable Future

The definition of decent work is generally inspired by the International Labour Organization's Agenda (ILO 2008) intended as a fundamental human right to aspire to. Specifically, the characteristics of decent work are the following: (a) opportunities to have a productive job that delivers a fair income, (b) safety in the workplace and social protection for families, (c) prospects for personal development and social integration, (d) freedom for people to express their concerns, organize, and participate in the decisions that affect their lives, and (e) equality of opportunity and treatment for all women and men.

The concept of decent work has been recently mentioned among the main priorities in the global consultations within the framework of the 2030 Sustainable Development (United Nations 2015a) to “promote sustained, inclusive, and sustainable economic growth, full and productive employment as well as decent work for all” (23).

The studies that examined the construct of decent work connected to the achievement of sustainable development in young people have detected significant relationships between the idea of decent and meaningful work, life satisfaction, and career adaptability (Nota et al. 2020; Santilli et al. 2023). Moreover, the idea of finding decent work was found to be a mediator of the relationship between perceived job instability and life satisfaction in a sample of Italian, Spanish, and Swiss young adults (Zammitti et al. 2023).

This approach considers work not only from an individual perspective but also from social and environmental points of view. It emphasizes the present while also being oriented toward the future, contributing to the well‐being of future generations (Massoudi et al. 2018). In this perspective, life design interventions are planned to help young people attain decent and meaningful work, promoting a complex idea of work not only for the benefit of the individual but inspired by less ego‐centric values, for the construction of life contexts and conditions that aim at sustainable development, acknowledging the environmental and social impact of professional actions (Ford and Smith 2020; Nota et al. 2020). For example, Maree (2018), sticking to the life design paradigm, implemented two career intervention projects for workers in developing countries that helped them develop stronger career resilience, and a sense of occupational, personal, and self‐identity, therefore increasing their chances of finding sustainable decent work.

## 3 Critical Consciousness

The concept of critical consciousness emerged in the 1920s and 1930s as a response to the social, political, and economic crises of the time. Developed within the theoretical framework of the Frankfurt School, it used an interdisciplinary approach to understanding society and emphasized the emancipation of dominated and oppressed classes. Max Horkheimer, in his seminal work “Traditional Theory and Critical Theory” (1937), critiqued contemporary society's capitalist and anthropocentric elements, envisioning a “situation without exploitation and oppression, in which humanity is genuinely aware of itself” (Horkheimer 1937, 36–37).

In the 20th century, Paulo Freire popularized the concept of critical consciousness, or conscientização, in “The Pedagogy of the Oppressed” (1970). Freire described it as the awareness of systemic marginalization across domains such as education, work, and social life, coupled with the drive to counteract this marginalization (Freire 1992).

Contemporary globalization has amplified forms of exploitation and oppression, necessitating renewed focus on critical consciousness (Manfredi and Reggio 2007). Critical consciousness entails evaluating power dynamics, dominant ideologies, and oppressive structures, empowering individuals to recognize privilege, address systemic inequalities, and engage in transformative action (Cadenas et al. 2018; Watts et al. 2011; Diemer et al. 2021).

Aligned with Freire's theories, modern frameworks view critical consciousness as comprising three elements: (1) *Critical reflection* involves the moral rejection of social inequities, such as economic and racial discrimination; (2) *Critical motivation* relates to the perceived ability to enact social and political change and (3) *Critical action* denotes commitment to activities addressing societal issues, from voting to peaceful protests (Cadenas and McWhirter 2022b; Watts et al. 2011).

Critical consciousness expands thought and action by addressing issues like human rights violations, environmental degradation, and systemic discrimination. Identifying underlying dynamics allows individuals to enact personal and collective changes for social justice (Nota et al. 2020; Jemal 2017; Mitchell et al. 2018). It is integral to personal growth and decision‐making processes, influencing collective life participation and professional choices (Kenny et al. 2019).

A review by Heberle et al. (2020) links critical consciousness to indicators of well‐being and empowerment, particularly for marginalized groups. Professionally, it correlates with adaptability, hope, resilience, and persistence, essential for navigating labor market complexities and pursuing sustainable career paths (Diemer et al. 2006; Duffy et al. 2016).

There are a limited number of interventions in the literature that aim to stimulate critical consciousness in young people in disadvantaged situations, with the aim of supporting them to face the challenges of society. Two of these are described below.

“Young Warriors” (Watts and Abdul‐Adil 1998) is an educational program aimed at African American adolescents, based on the theories of Paulo Freire, with the goal of promoting the development of critical consciousness and analysis of processes of oppression. Structured in eight 40‐min sessions, cultural materials such as music videos and films are employed to stimulate critical thinking in participants and foster an in‐depth understanding of social inequalities. Through the integration of elements of African and African American culture, the program encourages critical thinking about identity and masculinity, as well as promoting active engagement for social change. At the conclusion of the program, an increase in critical thinking skills and consciousness, as well as their active involvement in the community, was observed in participants. Another relevant critical consciousness program is Casanova's “Movimiento La Libertad” (MLL) (2024). This is an extracurricular program for Latinx adolescents aimed at developing critical consciousness and activism against educational injustices and anti‐immigrant policies. Based on agencies of transformational resistance framework, the program offers weekly 2‐h meetings with a curriculum focused on Latinx history, culture, and social realities. Through liberating pedagogical practices, such as sharing circles and the involvement of co‐conspiring adults, participants are encouraged to think critically and act against oppression. The program fosters a sense of belonging and authentic relationships between students and facilitators, strengthening leadership and civic engagement. Results demonstrate an increase in social awareness and ability to address inequality.

Career guidance plays a pivotal role in fostering critical consciousness among young people, encouraging reflection on global challenges and their alignment with educational and professional goals (Cadenas and McWhirter 2022b). In line with this, Life Design aims to support youth in acquiring critical consciousness about inequality, professional barriers, and social responsibilities, aligning career choices with broader ecological and societal objectives (Cadenas and McWhirter 2022a).

## 4 Research Goals and Hypotheses

The primary goal of this study is to evaluate the efficacy of a career intervention designed to enhance high school students' career adaptability, perception of decent work, and critical consciousness within the context of global socio‐political and environmental challenges. Specifically, the career intervention aims to equip students with the cognitive and emotional tools necessary for understanding systemic issues such as inclusion, sustainability, and social justice, while also fostering key career‐related competencies.

The study involved students in the final year of high school (12th grade in Italy, equivalent to the 5th year of upper secondary education), whose classes were randomly assigned to an intervention or a no‐treatment control group. In alignment with these objectives, the study will test the following hypotheses: (a) Students in the intervention group will show greater improvements in career adaptability (concern, curiosity, collaboration) than those in the control group; (b) They will report a more future‐oriented and socially aware perception of decent work; (c) They will exhibit higher levels of critical consciousness; (d) They will demonstrate enhanced career decisiveness, internal locus of control, and ability to gather information; (e) These gains will be sustained at 6‐month follow‐up.

Moreover, this study will assess participant satisfaction in the career intervention, hypothesizing that participants of the intervention group would perceive its usefulness and significance.

## 5 Methods

### 5.1 Participants

The participants were 266 high school students (average age = 17.20 years, SD = 0.50), assigned to either an intervention group or a no‐treatment control group. The intervention group included 145 students (54.5%; 52 male, 93 female) from seven classes across two schools: 100 students from Scientific High School (5 classes) and 45 from Classical High School (2 classes). All students were in the 12th grade (the final year of upper secondary education in Italy), with an average age of 17.00 years (SD = 0.63).

The no‐treatment control group was composed of 121 students (45.5%; 25 male, 96 female) from seven classes across three different schools, which did not overlap with those in the intervention group. This group included 92 students from Scientific High School (5 classes), 19 from Classical High School (1 class), and 10 from Art High School (1 class). Their average age was 17.61 years (SD = 0.56).

All students were enrolled in public high schools located in urban and semi‐urban areas of northern Italy. The educational tracks differed by curricular focus: classical high schools emphasized humanities and philosophy, scientific high schools focused on STEM subjects, and art high schools specialized in visual and applied arts. Data on race/ethnicity were not collected due to national legal and cultural constraints, but the socioeconomic background of participants—based on school profiles—ranged from lower‐middle to middle class.

### 5.2 Measures

#### 5.2.1 Career Adaptability

We used the Career Adapt‐Abilities Scale‐Italian Form (Soresi et al. 2012). The scale includes 35 items set on a 5‐point Likert‐type scale ranging from 1 (not strong) to 5 (strongest). The items form five separate subscales that measure the career adaptability resources of Concern, Control, Curiosity, Confidence, and Collaboration. The first four subscales (24 items) also combine to yield a total career adaptability score. Some examples of items are “I think about my future and try to predict it” (Concern); “I can resist provocations” (Control); “I like exploring, fantasizing, imagining, thinking in an unconventional way, finding novel solutions” (Curiosity); “I am confident in my abilities to take good decisions” (Confidence); and “I collaborate and do not hesitate to do my part” (Cooperation). Prior research (Soresi et al. 2012) has demonstrated that this instrument exhibits robust psychometric properties (i.e., internal consistency reliability, content, and construct validity). In the present study, the Cronbach's alphas for the five subscales were 0.84 (concern), 0.81 (control), 0.83 (curiosity), 0.85 (confidence), 0.80 (cooperation) for the intervention group, and 0.84 (concern), 0.80 (control), 0.80 (curiosity), 0.83 (confidence), 0.82 (collaboration) for the no‐treatment control group.

#### 5.2.2 Perception of Decent Work

We used the Decent Work for Inclusive and Sustainable Future Construction Scale (Zammitti et al. 2023) that assesses the perception of decent work from a broad perspective that looks at inclusion and sustainability. It consists of six items set on a 5‐point Likert scale ranging from 1 (Definitely NO, this is not what I think about the work I will do) to 5 (Definitely YES, this is what I think of the work I will do). The items support a vision of decent work as an opportunity to contribute to building inclusive and sustainable societies. Higher scores indicate a stronger idea of decent work oriented towards the future and not just in the present. An example of an item is “People must not accept work in a place that does not grant everybody the right to also freely express their opinions about the work performed”. The study of Zammitti et al. (2023) showed good internal consistency, reliability, and content and construct validity. In the present study, Cronbach's alpha was 0.75 for the intervention group and 0.73 for the no‐treatment control group.

#### 5.2.3 Critical Consciousness

We used the questionnaire “Critical Consciousness for the Development of Inclusive and Sustainable Futures” for young adolescents and adults (Santilli et al. 2025). To stimulate self‐assessments, respondents were asked to answer a total of 84 items using a 5‐point Likert scale (1 = “not at all” and 5 = “very much”). Examples of items include: “I believe that discrimination is not a problem in my country,” “I feel uncomfortable seeing the state of poverty in which some people live,” and “During the last 4 weeks, I have consumed local products”. The factor structure was confirmed through confirmatory factor analysis (CFA), which demonstrated excellent model fit: *χ*²(1854) = 3572.91, *p* < 0.001; comparative fit index (CFI) = 0.972; Tucker–Lewis Index (TLI) = 0.963; root mean square error of approximation (RMSEA) = 0.043, 90% CI [0.041, 0.045]; and standardized root mean square residual (SRMR) = 0.045.

The study conducted by Santilli et al. (2025) also reported satisfactory psychometric properties, including internal consistency and construct and content validity. In the present study, Cronbach's alpha was 0.92 for the intervention group and 0.95 for the no‐treatment control group, indicating excellent internal reliability.

#### 5.2.4 Career Decisiveness, Internality and Ability to Collect Information

We used the questionnaire “Ideas and Attitudes on School‐Career Future” (Soresi and Nota 2003) to assess career decidedness. This instrument is composed of 16 items in which participants indicate on a 7‐point Likert scale (1 = “It doesn't describe my point of view at all”; 7 = “It describes perfectly my point of view”) how much they feel described by affirmations presented to them. This questionnaire evaluates three dimensions: decisiveness and confidence in one's academic and professional future; internality; and the ability to gather information relevant to one's choice. Some example items are “I have decided what I want to do and I am confident that I have chosen well,” “I know how to gather the information I need,” and “I think I know my career aspirations well.” This instrument, as validated by Soresi and Nota (2003), has been employed in a number of studies, wherein its efficacy in the analysis of career decidedness in high school students and in verifying the efficacy of sustainable career guidance interventions has been demonstrated (e.g., Ginevra et al. 2016; Santilli et al. 2020). In the present study, the Cronbach's alphas for the three subscales were 0.94 (decisiveness), 0.81 (internality), 0.70 (collecting information) for the intervention group, and 0.95 (decisiveness), 0.79 (internality), 0.64 (collecting information) for the no‐treatment control group.

#### 5.2.5 Intervention Acceptability and Participant Satisfaction

To examine the participant satisfaction of the career intervention, we created five items that were designed for use posttest to determine the intervention group participants' global satisfaction with the intervention, and the perceived usefulness and significance of the training and actions based on researches by Santilli et al. (2019, 2022). The participants responded to each item using a 5‐point Likert scale ranging from 1 (not satisfied/not at all) to 5 (extremely satisfied/totally). Some examples of items are “How satisfied are you with the project you carried out at school?” and “Would you recommend any of your fellow students to follow this career guidance programme?” In the present study, the Cronbach's alpha is 0.91.

### 5.3 Procedure

Following Shadish (2002), to explore the hypotheses of the study, we created a quasi‐experimental research with two repeated measures taken in an intervention group and a no‐treatment control group. All participants were involved through contacting high schools. A detailed description of the career intervention was circulated to schools that had expressed interest. Subsequently, these schools were requested to indicate their interest in participating. The schools had at least the final year of high school (equivalent to the 5th year of upper secondary education) with no more than 20 students. Five high schools showed interest in participating in the study, for a total of fourteen high school classes, seven in the intervention group and seven in the no‐treatment control group. While the intervention group was taking part in the career intervention, the no‐treatment control group continued with normal schoolwork. Immediately prior (pretest) and following (posttest) the intervention, participants of both intervention and no‐treatment control groups were assessed the pre and posttest measures. At the end of the intervention, all students in the intervention group also completed a measure of social validity pertaining to the career guidance intervention. Furthermore, 33 participants in the intervention group (22.8%) were able to complete the pre and posttest measures 6 months after the career intervention (follow‐up) to evaluate its long‐term impact.

### 5.4 Ethics Statement

All phases of the research project were developed and implemented in accordance with the ethical and deontological standards provided by the Italian Society of Vocational Guidance (SIO) and the Code of Ethics of the Italian Association of Psychology (AIP; revised 2022), which is inspired by the principles of the Declaration of Helsinki (1964/2013). The study protocol was approved by the Ethics Committee of the Department of FISPPA, University of Padova, Italy (Project identification code: 814‐b). Informed consent was obtained from all participants and, when applicable, from their parents or legal guardians. Participation was voluntary, anonymity and confidentiality were guaranteed, and participants were informed of their right to withdraw from the study at any time without negative consequences.

### 5.5 “Knowing the Present to Imagine a Sustainable and Inclusive Future” Career Intervention

For the intervention group, we developed and carried out a career intervention for high school students. The title of the career intervention is “Knowing the present to imagine a Sustainable and Inclusive Future." It is comprised of 5 weekly didactic units, each with a duration of 2 h and conducted during school hours. Each didactic unit is delivered by career guidance professionals who hold postmaster's diploma certifications. The career intervention was implemented in small groups of 8–10 classmates to guarantee personalized reinforcements and feedback to participants and to provide them with more opportunities to actively participate, by asking and answering questions.

The goal of the career intervention was to foster career adaptability and perception of decent work in participants involved and to increase critical consciousness about the contextual processes that produce and encourage the exploitation of the planet and human beings, who are viewed solely as “capital” and “labor forces.” To achieve this, strategies such as facilitated discussions, reflective practices, and participatory activities were implemented to promote critical reflection and engage individuals in examining the underlying power dynamics and ideologies that perpetuate such exploitation. The career intervention also aimed to foster decisiveness and confidence in one's academic and professional future, internality, and the ability to gather information relevant to one's choice.

As regards the didactic units developed, the first unit focused on explaining the meaning of career guidance and the types of assistance it provides to individuals. To enhance a positive concern about the future, a sense of control, curiosity, and confidence, participants were guided in reflecting on their hopes and concerns for their professional future. After collecting adolescents' expectations, a guided reflection on the concept of work was conducted, presenting it as an evolving element that changes over time, influenced by contextual phenomena. Specifically, career guidance professionals conducted a critical reflection about the presence of social inequalities and environmental issues, to foster a critical knowledge about these topics.

The second and third didactic units aim to promote decisiveness and confidence in one's academic and professional future, internality, and the ability to gather information. During these units, the discussions were focused on issues related to discrimination in work contexts and environmental pollution because of indecent working conditions, stimulating emotional responses to these issues.

Specifically, the second didactic unit aimed to develop a global perspective on globalization. The concept of globalization was presented, highlighting its advantages and disadvantages in terms of its impact on the world of work. After discussing the negative effects of globalization, students were encouraged to reflect on the importance of defending rights to counteract these processes. They were asked to discuss in small groups about rights associated with decent work (i.e., Article 23 of the Universal Declaration of Human Rights, 1948; Article 4 of the Italian Constitution; Article 1 of the United Nations Declaration on the Human Environment), the benefits of upholding these rights and the potential consequences of their violation, such as exploitation and reduced job opportunities. To foster a critical reflection, some prompt questions were asked, for example: “How globalization can undermine the protection of the right to work?” Then, the adolescents were asked to share their thoughts with their peers.

The third didactic unit focused on technological progress. After presenting the concept of technological advancement, a guided reflection was conducted on technological development and its impact on the labor market, discussing its pros and cons. Students were prompted to consider the importance of defending rights in this context. Specific rights related to technology use and employment (i.e., Article 8 of the European Convention on Human Rights) were presented. Then, the adolescents explored the advantages of maintaining these rights and the consequences of their violation, such as exploitation and reduced employment opportunities. They discussed rights related to technological advancement and their consequences in small groups, with prompt questions like: “What benefits might these rights give to your future?” Following the discussion, adolescents were invited to share their thoughts with their peers.

In the fourth didactic unit, the theme of sustainability was addressed, focusing on how professions can be inclusive and sustainable. After providing the definitions of inclusion and sustainability, the career guidance professional recalled key concepts from previous units (i.e., work, globalization, technological development), with the aim of fostering perception of decent work for an inclusive and sustainable career construction. A guided reflection on inclusion and sustainability was conducted, emphasizing how education and critical thinking can be valuable resources in achieving the UN Sustainable Development Goals (United Nations 2015b), to improve a critical consciousness about social inequalities and environmental issues. Students were asked to reflect on the benefits of fostering inclusive and sustainable professions, including respect for individual and collective rights, environmental stewardship, and the promotion of decent work. Using images and videos, the career guidance professional provided some examples of sustainable career activities. Then, the adolescents were invited to give other examples.

The final didactic unit provided a guided reflection on students' professional futures, to foster activism and participation in social and environmental change, and intention to collaborate with others in their professional future. The goal was to support them in designing their “mission possible,” considering current challenges and threats as well as the SDGs (United Nations 2015b). The “mission possible” consisted in a synthesis about their learnings from the intervention, identifying key goals for their professional aspirations and potential activities to achieve these goals. The participants were asked to focus on two or three goals of the 2030 Agenda for sustainable development and identify sustainable professional activities to achieve sustainable developmental goals (see Table 1).

**Table 1:** Structure of the “Knowing the Present to Imagine a Sustainable and Inclusive Future” career intervention.

Didactic unit	Aims	Main activities	Duration
**Unit 1—Meaning of work and the future**	Promote career concern, control, curiosity, and confidence; introduce career guidance and global challenges	Guided reflection on hopes and fears about the future; discussion on the evolving meaning of work; introduction to social inequalities and environmental issues	2 h
**Unit 2—Globalization and decent work**	Enhance decisiveness, internality, and awareness of labor rights	Presentation of globalization processes; group discussions on decent work rights (e.g., Universal Declaration of Human Right, Italian Constitution); reflection on consequences of rights violations	2 h
**Unit 3—Technological development and work**	Foster critical thinking about technology and employment; strengthen decision‐making skills	Analysis of technological progress and its impact on work; discussion of rights related to technology use; small‐group discussions and plenary sharing	2 h
**Unit 4—Inclusion, sustainability, and professions**	Promote perception of decent work for inclusive and sustainable futures; enhance critical consciousness	Definitions of inclusion and sustainability; reflection on UN Sustainable Development Goals (SDGs); examples of inclusive and sustainable professions using images and videos	2 h
**Unit 5—Designing a sustainable professional future**	Foster collaboration, social participation, and future‐oriented career planning	Development of a personal “mission possible”; identification of professional goals aligned with selected SDGs; reflection on sustainable career actions	2 h

### 5.6 Data Analysis

The measures were administered via a Google module. Answers to the questions were mandatory to complete the protocol. Therefore, there was no missing data. All analyses were conducted using IBM SPSS Statistics for Windows, Version 26 (IBM Corp 2019). Preliminarily, we checked if the intervention group and the no‐treatment control group were homogeneous for socio‐demographic characteristics and, at the pretest stage, the two groups were compared on all study variables. Specifically, we performed the *χ* ^2^ test for gender and *t‐*tests for age and dependent variables (i.e., concern, control, curiosity, confidence, collaboration; perception of decent work; critical consciousness; decisiveness and confidence in one's academic and professional future, internality, ability to gather information relevant to one's choice;). We also performed Pearson correlations between all dependent variables. In examining the correlations, we classified effect sizes as small (0.10–0.25), medium (0.25–0.40), or large (over 0.40) (Cohen 1988).

The career intervention's efficacy was valued by carrying out repeated‐measures Linear Mixed Models (LMM). LMM are an extension of standard linear regression that allows to take account of correlations within repeated measures by specifying that residual errors are correlated (Cnaan et al. 1997; Meteyard and Davies 2020). We run repeated‐measures LMM with treatment (intervention vs. no‐treatment control group) and Time (pretest vs. posttest measurement) as fixed effects, and individual variance (participant) and classes of adolescents as random effects. The Treatment was the between‐group variable, and the Time was the within‐group variable.

To determine whether the effects of the career intervention were sustained over time, a paired‐samples *t*‐test was performed comparing the post‐test scores with the follow‐up scores. This test was chosen in line with suggestions by Field (2013) in comparing two related samples or measurements taken at different times.

## 6 Results

### 6.1 Preliminary Analyses

No significant gender differences were detected between the intervention group and the no‐treatment control group: *χ* ^2^(1) = 1.442, *p* = 0.230; nor were any age differences: *t* (264) = 1.123, *p* = 0.262.

At the pretest stage, intervention and no‐treatment control groups showed a significant difference on five factors: concern (*t*_(262)_ = 3.069, *p* = 0.002, Cohen's *d* = 0.19); control (*t*_(264)_ = 2.958, *p* = 0.003, Cohen's *d* = 0.0270); critical consciousness (*t*_(262)_ = 1.791, *p* = 0.037, Cohen's *d* = 0.21); decisiveness and confidence in one's academic and professional future (*t*_(264)_ = 3.958, *p* < 0.001, Cohen's *d* = 0.41), and ability to gather information related to one's choice (*t*_(264)_ = 3.071, *p* = 0.002, Cohen's *d* = 0.30). Specifically, the intervention group started with lower levels than the no‐treatment control group on career adaptability, critical consciousness, decisiveness, and the ability to collect information. Table 2 reports means and standard deviations of the dependent variables of both intervention and no‐treatment control groups at pre and posttest.

**Table 2:** Means (M) and standard deviations (SD) of experimental group and control group at pre and posttest.

	Pretest				Posttest			
	Experimental group		Control group		Experimental group		Control group	
	M	SD	M	SD	M	SD	M	SD
Concern	20.95	4.67	21.98	4.73	21.26	4.64	21.96	4.44
Control	23.57	4.42	23.76	4.04	23.03	4.68	23.79	4.29
Curiosity	22.45	4.11	22.57	4.30	22.75	5.36	22.81	4.12
Confidence	23.11	4.04	23.12	4.06	23.92	4.49	23.25	4.35
Collaboration	40.27	6.80	40.77	6.94	42.24	7.54	40.48	6.89
Perception of decent work	23.04	3.75	23.31	3.93	23.70	4.25	22.93	3.67
Critical consciousness	299.52	30.52	308.70	39.00	316.70	31.68	309.29	39.03
Decisiveness and confidence	39.96	14.88	46.16	15.13	43.18	14.52	46.22	14.77
Internality	19.81	4.73	20.45	4.59	21.82	4.40	19.53	4.87
Ability to gather information	7.91	3.15	8.91	2.96	8.35	2.82	9.02	2.79

Correlations between measures collected from both intervention and no‐treatment control groups at pretest are reported in Table 3. We can observe significant positive correlations between career adaptability, perception of decent work for an inclusive and sustainable career construction, critical consciousness of issues and threats related to inclusion and sustainability, and decisiveness and confidence about one's academic and professional future. Moreover, significant positive correlations were found between perception of decent work for an inclusive and sustainable career construction and collaboration, and the dimensions of concern, curiosity, and control within career adaptability. Overall, all Pearson's *r* values of significant correlations indicate a small‐to‐medium effect size of the associations between variables, except for perception of decent work, which show a large effect size with critical consciousness.

**Table 3:** Correlations between dependent variables for all participants at pretest.

	1	2	3	4	5	6	7	8	9	10
1	1	0.553**	0.514**	0.524**	0.270**	0.210**	0.246**	0.631**	0.417**	0.298**
2		1	0.669**	0.692**	0.323**	0.111	0.257**	0.395**	0.342**	0.286**
3			1	0.642**	0.448**	0.175**	0.330**	0.323**	0.248**	0.212**
4				1	0.450**	0.131*	0.311**	0.270**	0.262**	0.081
5					1	0.246**	0.363**	0.109	0.050	0.036
6						1	0.497**	0.056	0.001	0.000
7							1	0.093	0.122*	−0.080
8								1	0.581**	0.545**
9									1	0.347**
10										1

* *p* < 0.05;

** *p* < 0.001.

*Note:* 1. Concern; 2. Control; 3. Curiosity; 4. Confidence; 5. Collaboration; 6. Perception of decent work; 7. Critical consciousness; 8. Decisiveness and confidence in one's academic and professional future; 9. Internality; 10. Ability to gather information related to one's choice.

### 6.2 Efficacy Evaluation

#### 6.2.1 Career Adaptability

Regarding career adaptability and collaboration, five repeated‐measures LMM were conducted. Significant treatment × time interactions were found for the following variables (see Table 4): Concern (*t*_(264)_ = −2.346, *p* = 0.020), Curiosity (*t*_(264)_ = −2.100, *p* = 0.037), Collaboration (*t*_(264)_ = 2.770, *p* = 0.006).

**Table 4:** Parameter estimates, standard errors, confidence intervals, and statistical significance in the LMM analyses of the experimental and control group in the pre and posttest for career adaptability resources.

Dependent variable	Effect	*β*	SE	95% CI	*t*	*p*‐value	Wald's *Z* for the random intercept for participants (*p*)	Wald's *Z* for the random intercept for classes (*p*)
Concern	Time	0.47	0.46	−0.43, 1.37	1.031	0.304	5.892 (< 0.001)	1.45 (0.136)
	Treatment	−0.27	0.56	−1.37, 0.84	−0.476	0.635		
	Time × treatment	−1.46	0.62	−2.68, −0.23	−2.346	0.020		
Control	Time	0.52	0.43	−0.32 1.36	1.223	0.222	5.479 (< 0.001)	1.841 (0.066)
	Treatment	−0.51	0.79	−2.14, 1.13	−0.644	0.526		
	Time × treatment	−0.65	0.58	−1.79 0.49	−1.130	0.260		
Curiosity	Time	0.26	0.45	−0.62, 1.13	0.575	0.566	5.324 (< 0.001)	1.502 (0.133)
	Treatment	0.39	0.72	−1.09, 1.87	0.543	0.592		
	Time × treatment	−1.27	0.60	−2.46, −0.08	−2.100	0.037		
Confidence	Time	0.29	0.40	−0.50, 1.08	0.719	0.473	5.584 (< 0.001)	−1.502 (0.134)
	Treatment	−0.02	0.73		−0.027	0.978		
	Time x treatment	−0.82	0.55	−1.90, 0.26		0.134		
Collaboration	Time	0.98	0.60	−0.21 2.17	1.626	0.105	7.140 (< 0.001)	0.379 (0.704)
	Treatment	0.73	0.89	−1.13, 2.60	0.820	0.422		
	Time × treatment	−2.28	0.82	−3.90, −0.66	2.770	0.006		

The analysis revealed that the average levels of concern, curiosity, and collaboration were higher in the posttest participants who took part in the career intervention than the average levels reported by the no‐treatment control group participants.

#### 6.2.2 Perception of Decent Work

Regarding the perception of decent work, a repeated‐measures Linear Mixed Model was conducted. A significant treatment × time interaction was found: *t*_(264)_ = −2.736, *p* = 0.007 (see Table 5).

**Table 5:** Parameter estimates, standard errors, confidence intervals, and statistical significance in the LMM analyses of the experimental and control group in the pre and posttest for ideas about decent work.

Dependent variable	Effect	*β*	SE	95% CI	*t*	*p*‐value	Wald's *Z* for the random intercept for participants (*p*)	Wald's *Z* for the random intercept for classes (*p*)
Perception of decent work	Time	0.39	0.38	−0.35, 1.13	1.030	0.304	6.094 (< 0.001)	1.264 (0.206)
	Treatment	0.89	0.62	−0.40, 2.19	1.432	0.167		
	Time × treatment	−1.40	0.51	−2.41, −0.39	−2.736	0.007		

The analysis revealed that the intervention group reported, at the posttest, stronger perception of decent work for an inclusive and sustainable career construction than the participants of the no‐treatment control group.

#### 6.2.3 Critical Consciousness

Regarding critical consciousness, one repeated‐measures LMM was conducted. A significant treatment × time interaction was found: *t*_(264)_ = −4.483, *p* < 0.001 (see Table 6).

**Table 6:** Parameter estimates, standard errors, confidence intervals, and statistical significance in the LMM analyses of the experimental and control group in the pre and posttest for critical consciousness.

Dependent variable	Effect	*β*	SE	95% CI	*t*	*p*‐value	Wald's *Z* for the random intercept for participants (*p*)	Wald's *Z* for the random intercept for classes (*p*)
Critical consciousness	Time	−0.59	2.73	−5.97, 4.80	−0.215	0.830	7.993 (< 0.001)	1.887 (0.059)
	Treatment	6.61	6.71	−7.41, 20.62	0.985	0.337		
	Time × Treatment	−16.60	3.70	−23.89, −9.31	−4.483	< 0.001		

The analysis revealed that students in the intervention group showed more critical consciousness than the no‐treatment control group.

#### 6.2.4 Decisiveness, Internality, and Ability to Gather Information

Regarding decisiveness and confidence in one's academic and professional future, internality and ability to gather information related to one's choice, three repeated measures LMM were conducted. A significant treatment × time interactions were found for decisiveness and confidence in one's academic and professional future (*t*_(264)_ = −3.008, *p* = 0.003), and Internality (*t*_(264)_ = −2.595, *p* = 0.010) (see Table 7).

**Table 7:** Parameter estimates, standard errors, confidence intervals, and statistical significance in the LMM analyses of the experimental and control group in the pre and posttest for decisiveness and confidence in one's academic and professional future, internality, and ability to gather information related to one's choice.

Dependent variable	Effect	*β*	SE	95% CI	*t*	*p*‐value	Wald's *Z* for the random intercept for participants (*p*)	Wald's *Z* for the random intercept for classes (*p*)
Decisiveness and confidence	Time	0.08	1.29	−2.46, 2.63	0.064	0.949	7.356 (< 0.001)	0.999 (0.318)
	Treatment	−1.94	2.16	−6.38, 2.50	−0.900	0.377		
	Time × treatment	−5.28	1.75	−8.73, −1.82	−3.008	0.003		
Internality	Time	1.04	0.45	0.15, 1.94	2.294	0.023	5.888 (< 0.001)	0.705 (0.481)
	Treatment	0.65	0.64	−0.67, 1.97	1.018	0.319		
	Time × treatment	−1.60	0.62	−2.82, −0.39	−2.595	0.010		
Ability to gather information	Time	−0.09	0.27	−0.62, 0.44	−0.337	0.737	6.539 (< 0.001)	1.604 (0.109)
	Treatment	−0.49	0.48	−1.48, 0.50	−1.018	0.318		
	Time × treatment	−0.53	0.37	−1.25, 0.19	−1.449	0.149		

The analysis revealed that the average levels of decisiveness and confidence in one's academic and professional future, and internality were higher in the posttest participants who took part in the career intervention than the average levels reported by the no‐treatment control group participants.

### 6.3 Intervention Acceptability and Participant Satisfaction

We assessed the social validity of the career intervention from the perspective of intervention group's participants. Overall, the students rated career intervention as being essential to their reflection upon specific phenomenon and their impact on the labor market, and appropriate to handle future professional projects. Specifically, 92% stated that they were satisfied with career intervention, 6% stated that they were neutral, and 2% stated that they were a little satisfied. Moreover, 92% found it useful to reflect about globalization, 96% found it useful to reflect about technological advancements, and 87% found useful to reflect about sustainable future growth. Finally, 87% would suggest participation in this career intervention to other classmates.

### 6.4 Follow‐Up

The results showed a significant improvement from post‐test to follow‐up in career adaptability for the intervention group. In particular, the findings suggest that there was a significant improvement from the posttest to follow‐up in career control with a moderate effect size (*t*_(32)_ = 2.424, *p* = 0.021, Cohen's *d* = 0.42), in career curiosity (*t*_(32)_ = 2.397, *p* = 0.023, Cohen's *d* = 0.43) with a moderate effect size, and in collaboration (*t*_(32)_ = 2.292, *p* = 0.029, Cohen's *d* = 0.40) with a moderate effect size. Career concern and career confidence did not present statistically significant changes over time.

Regarding critical consciousness, there was a significant increase from post‐test to follow‐up (*t*_(32)_ = −2.199, *p* = 0.035) for the intervention group. The effect size is large (Cohen's *d* = 0.81), indicating a substantial difference.

As regards perception of decent work, the analyses demonstrated a significant increase from post‐test to follow‐up. Specifically, the *t*‐test revealed a substantial improvement with a large effect size (*t*_(32)_ = 7.832, *p* = 0.001, Cohen's *d* = 4.690) for the intervention group, indicating a strong enhancement in perception of decent work for an inclusive and sustainable career construction.

Finally, the follow‐up results also showed a significant decrease in career decisiveness from post‐test to follow‐up (*t*_(32)_ = 2.425, *p* = 0.021, Cohen's *d* = 0.422) for the intervention group, suggesting a meaningful enhancement in this dimension over time. Ability to gather information related to one's choice presented an improvement, although the effect size was moderate (*t*_(32)_ = 1.955, *p* = 0.050, Cohen's *d* = 0.340).

## 7 Discussion

This study aimed to develop and evaluate the efficacy of a career intervention designed to enhance high school students' career adaptability, perception of decent work, critical consciousness, decisiveness, and confidence in one's academic and professional future, and internality, in the context of global and ecological challenges. The findings offer valuable insights into the impact of such a program on students' career‐related attitudes and behaviors.

This study contributes to the field by demonstrating how life design principles can be meaningfully integrated with the PWT to promote youth empowerment. The explicit inclusion of critical consciousness and decent work provides a model for bridging individual agency and structural awareness, a gap highlighted in prior literature (Medvide et al. 2019; Kenny et al. 2019).

The novelty of the program lies in its ability to integrate themes of social justice and sustainability into career education. This approach represents a significant shift from traditional practices and opens up new possibilities for reflecting on how work can contribute to building more equitable and sustainable communities.

Rather than simply enhancing career‐related skills, career intervention raises fundamental questions about how young people can prepare for a rapidly changing labor market while adopting a broader and more integrated perspective.

Participants showed significant improvements in career adaptability dimensions such as concern, curiosity, and collaboration, consistent with Savickas et al.'s (2009) emphasis on these factors in career construction theory. These improvements suggest enhanced preparedness for future career transitions, a crucial aspect in today's dynamic job market (Rudolph et al. 2017).

The intervention successfully broadened students' understanding of decent work to include social and environmental considerations, reflecting recent calls for integrating sustainability into career intervention (Guichard 2022; Nota et al. 2020). This expanded perception aligns with the growing recognition of work's role in addressing global challenges and promoting sustainable development (Massoudi et al. 2018).

Notably, the career intervention significantly increased students' critical consciousness, supporting Watts et al.'s (2011) and Diemer et al.'s (2021) assertions about the importance of critical awareness in career development. This outcome is particularly innovative, as it represents one of the first empirical demonstrations of how critical consciousness can be effectively integrated into a life design‐based career intervention for high school students.

The observed enhancements in decisiveness and internality suggest improved career planning skills, consistent with life design principles (Savickas et al. 2009) and recent work on career decision‐making self‐efficacy (Lent and Brown 2013). These improvements indicate that students are better equipped to make informed career choices in complex, rapidly changing environments.

The positive social validity results further underscore the relevance and effectiveness of this integrated approach, suggesting its potential for wider application in career intervention programs. This aligns with recent calls for more holistic, context‐sensitive career interventions (Pouyaud and Guichard 2017) and demonstrates the feasibility of implementing such approaches in high school settings.

The follow‐up analysis revealed sustained and, in some cases, enhanced effects of the career intervention group over time, providing compelling evidence for its long‐term impact. Significant improvements were observed in several key areas, including career adaptability, perception of decent work, and critical consciousness. These findings not only align with existing literature on the importance of these factors for career success and well‐being (Judge and Bono 2001) but also extend our understanding of the durability of career interventions.

The persistence and even enhancement of effects at follow‐up are particularly noteworthy. It suggests that the intervention may have initiated a process of ongoing reflection and development among participants, rather than merely providing a temporary boost. This aligns with the Life Design paradigm's emphasis on continuous adaptation and growth (Savickas et al. 2009).

Overall, these findings contribute significantly to the emerging field of career intervention that integrates social justice and sustainability (Cohen‐Scali et al. 2018; Cadenas and McWhirter 2022a). The career intervention's innovative approach, which combines critical consciousness activities with traditional career development strategies, offers a new paradigm for preparing students to navigate complex global challenges while pursuing meaningful and sustainable careers.

In the context of this article, these results provide empirical support for the efficacy of incorporating critical consciousness and sustainability concepts into career guidance programs. They demonstrate that such an approach can effectively prepare young people for the complexities of modern career landscapes while fostering a sense of social responsibility and global awareness.

### 7.1 Limitations and Future Directions

Despite the promising results, there are several important limitations to consider. One significant limitation is related to the generalizability of the findings. The career intervention was conducted within a specific context, namely, high schools in northern Italy. This regional focus may limit the applicability of the results to other geographical locations or cultural settings. The necessity for educational and career guidance varies considerably across different regions and countries, influenced by a variety of social, economic, and cultural factors. Thus, the efficacy of the career intervention in other contexts remains uncertain, and future research should aim to replicate the study in different settings to determine whether the observed outcomes can be generalized beyond the Italian context (Glatthorn and Joyner 2005).

Another limitation is the study design, which, while valuable, may limit the robustness of the conclusions drawn. While the study employed a pretest and posttest approach, it did not use a fully randomized controlled trial, which is considered the gold standard for evaluating causal relationships (Gordon 2009). The presence of pretest differences between the experimental and control groups suggests that initial disparities in career‐related attitudes and knowledge might have influenced the results. Although statistical adjustments were made, the design still falls short in isolating the specific effects of the intervention. Future studies should prioritize randomized controlled trials to strengthen causal inferences and mitigate biases, thereby providing stronger evidence for the program's effectiveness (Field 2013).

The limited diversity of the sample also represents a constraint. The study was conducted with a relatively small number of schools and participants, which restricts the capacity to conduct more complex statistical analyses, such as mixed‐effects models, to account for variability between different schools and regions. The sample size and lack of diversity may affect the generalizability of the findings to other educational environments. A larger and more diverse sample would enable researchers to better understand the career intervention's impact across different demographic and institutional contexts, while also identifying moderating variables that could influence outcomes (Field 2013).

Another limitation concerns the short‐term focus of the evaluation. While the program demonstrated efficacy in improving career adaptability, perception of decent work, critical consciousness, decisiveness, and confidence in one's academic and professional future, and internality, the long‐term impact remains uncertain. Follow‐up results were limited to a small group, and more longitudinal studies are needed to assess the sustainability of these outcomes over time. Understanding whether the observed improvements translate into tangible career success, satisfaction, and resilience in real‐world settings would provide a more comprehensive picture of the career intervention's long‐term value (Schoon and Silbereisen 2009). Additionally, follow‐up results highlighted areas where further intervention may be needed. For instance, dimensions such as concern and confidence did not show significant changes, indicating that the career intervention might benefit from additional components to address these aspects effectively.

Moreover, the career intervention's regional specificity highlights the need for contextual adaptations. For instance, in areas facing acute environmental crises, the career intervention could emphasize ecological sustainability, whereas in regions with pronounced social inequalities, a greater focus on inclusion and equity might be more relevant. Tailoring the program to address these specific challenges would enhance its relevance and impact in diverse settings (Glatthorn and Joyner 2005).

Lastly, the study relies heavily on self‐reported measures, which may introduce response biases. While efforts were made to ensure the reliability of the instruments used, self‐reports can be influenced by social desirability and participants' subjective perceptions (Field 2013). Future research could incorporate additional objective measures, such as behavioral assessments or long‐term career tracking, to provide a more holistic evaluation of the career intervention's impact. An additional limitation of the present study concerns the absence of specific measures assessing identity development. Although identity construction is a core component of the Life Design paradigm, the current evaluation focused on career adaptability, perceptions of decent work, and critical consciousness, without directly examining changes in vocational identity. Future research could address this limitation by incorporating additional and more objective indicators of identity development, such as behavioral or narrative assessments of vocational identity. The integration of qualitative or mixed‐method approaches could provide a deeper understanding of how life design–based interventions support identity construction over time.

Despite these limitations, the findings underscore the potential of innovative career interventions to address critical global challenges and support students in their career development, paving the way for future research to build on and refine this approach.

### 7.2 Implications for Practice

The findings have significant practical implications for career interventions in high schools. Integrating critical consciousness and sustainability into career education can substantially enhance students' ability to cross the increasingly complex career landscape and make informed decisions that align with broader social and environmental goals. According to Savickas (2005), incorporating elements of social justice and sustainability into career education helps students develop a sense of purpose and alignment with their values, which is crucial for achieving long‐term career satisfaction and effectiveness.

Educators and career counselors should consider incorporating similar interventions to foster a more holistic approach to career development. By addressing not only individual career aspirations but also the global and ecological impacts of career choices, these interventions can help students develop a comprehensive understanding of how their professional paths can contribute to societal and environmental well‐being (Guichard 2022). As noted by Hirschi (2012), such integrative approaches can enhance career adaptability and preparedness, equipping students with the skills needed to thrive in a rapidly changing job market while also contributing positively to their communities.

## 8 Conclusion

In conclusion, this study provides robust evidence of the efficacy of a career intervention program in enhancing career adaptability, perceptions of decent work, critical consciousness, decisiveness, and confidence in one's academic and professional future, and internality among high school students. The career intervention's focus on addressing global and ecological challenges through career guidance is aligned with contemporary career development theories that emphasize the importance of integrating personal values with societal needs (Brown and Lent 2006; Nauta 2010). This intervention model, by fostering both agency and critical awareness, may serve as a replicable framework for career education programs aiming to empower youth as change agents in a globalized, uncertain world.
